# Time to classify: a narrative and scoping review of the old and the new classifications of perianal Crohn’s disease

**DOI:** 10.1007/s10151-025-03161-z

**Published:** 2025-05-26

**Authors:** T. Pelly, E. Anand, L. Hanna, E. Shakweh, S. Joshi, P. Lung, A. Hart, P. Tozer

**Affiliations:** 1 Imperial College London, London, UK; 2 St Mark’s The National Bowel Hospital, Central Middlesex, Acton Lane, London, UK

**Keywords:** Classification, Perianal Crohn’s disease, Phenotype, Fistula

## Abstract

Perianal Crohn’s disease (pCD) is a complex manifestation of Crohn’s disease. Classifying this patient cohort for both clinical purposes and for inclusion into research trials is challenging but crucial in order to improve outcomes. This review provides an overview of historical classifications of both fistulising and non-fistulising pCD, including the Park’s, Cardiff–Hughes and American Gastroenterological Association (AGA) classifications, as well as recent advances including the Treatment Optimisation and CLASSification of perianal Crohn’s disease (TOpClass) classification of fistulising pCD. Secondly, this article provides a scoping review of recent trials in pCD and describes how the cohorts in these trials relate to the TOpClass classification. Of the 19 studies relating to pCD that were identified, four could be confidently classified as class 2a. Seven could be classified as class 2a or 2b, but it was not possible to subdivide further, and seven to class 2a, 2b or 2c, but it was not possible to subdivide further. One study population was classified as class 2a or 2c. In eight studies, it was not specified whether patients with a defunctioning stoma were included or excluded. This review demonstrates the heterogeneous nature of some patient cohorts in previous clinical trials, and how the TOpClass classification may be used to group patients more accurately for clinical use and inclusion in research trials.

## Introduction

The importance of classifying disease has been recognised since ancient times, where treatises in the *Hippocratic Corpus* described the categorisation of diseases. In these texts, diseases were described in the *ad capite ad calcem* style, meaning from the top to the bottom of the body, in anatomical order [1]. The importance of the classification of diseases has long since been a foundation of modern medicine, and the ability to categorise with greater precision has allowed clinicians to select tailored treatments and improve patient outcomes.

Although Hippocrates began his classifications of diseases at the top of the body, it is also at the ‘bottom’ that classification is particularly important. Perianal manifestations of Crohn’s disease are common, with 26% of patients with Crohn’s disease developing a fistula within the first 20 years from diagnosis [2]. This particular phenotype can result in complex disease and encompasses marked variation in severity, anatomy, and responsiveness to medical and surgical treatments [3].

Classifying patients with pCD for treatment selection and inclusion in clinical trials is difficult and has taken different forms ever since it was first described by Penner and Crohn [4]. Perhaps the most widely used include Park’s classification [5] and the American Gastroenterological Association (AGA) definitions of simple and complex fistulising disease [6]. These classification systems focus predominantly on anatomical features. Classification systems to categorise non-fistulising features of perianal Crohn’s disease such as strictures, fissures, ulcers and skin tags have also been described. The most ubiquitous of these is the Cardiff–Hughes classification [7].

Recently, the Treatment Optimisation and CLASSification of perianal Crohn’s disease (TOpClass) consortium of experts in pCD developed a novel classification system for fistulising pCD, designed to focus less on anatomical and morphological elements. Instead it classifies disease according to distinct stages of severity requiring different treatment approaches and is based around patient goals [8].

This review article describes both historical and recent advances in the classification of fistulising pCD. We re-evaluate recent clinical trials relating to the treatment of pCD and relate these to the TOpClass classification. Finally, this article discusses the classification of non-fistulising pCD.

## The classification of perianal Crohn’s disease

### Historical classifications of fistulising perianal Crohn’s disease

**Fig. 1:**
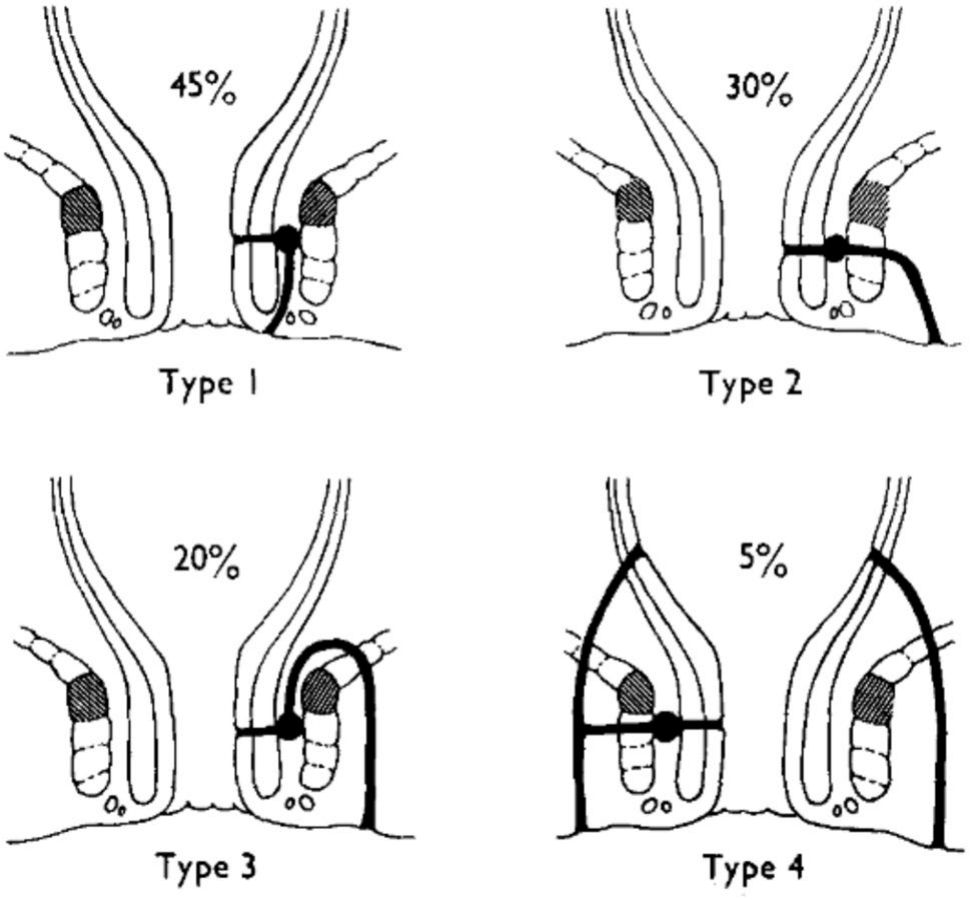
Parks classification (from Parks et al.) [5]. Type 1 is intersphincteric, type 2 is transphincteric, type 3 is suprasphincteric, and type 4 is extrasphincteric

The need for a novel clinically relevant classification system was identified in guidelines developed by an expert consensus process in 2014 [9]. A systematic review, later updated by Geldof et al., identified 18 classification systems relating to fistulising pCD [8]. The majority of these systems describe fistulae on the basis of their anatomy or disease activity. The most commonly used anatomical or morphological classifications are the Parks, Cardiff–Hughes and American Gastroenterological Association (AGA) classifications. The seminal Park’s classification, published in 1976, classifies fistulae into intersphincteric, transsphincteric, suprasphincteric and extrasphincteric anatomical positions [5]. This classification system, which was developed from analysis of a large cohort of patients treated surgically, was modified in 2001 to include submucosal fistulae [10]. This terminology remains ubiquitous in both clinical and research settings (Fig. 1).

The Cardiff–Hughes classification (Table 1), developed in 1978, classifies pCD according to three categories of disease morphology: ulceration, fistula/abscess and stricture. Fistulising disease is graded on a scale of 0–2 on the basis of the anatomical location (high/low) and anatomy (superficial and complex) [7]. This approach is analogous to the Montreal classification for luminal inflammatory bowel disease (IBD), defining the anatomical extent and nature of the disease [11]. The 2003 AGA classification describes fistulae as simple or complex on the basis of the anatomical level at which the sphincter is involved, number of external openings, associated abscesses or proctitis [6].

**Table 1:** Classification systems

Classification					
Cardiff–Hughes Classification (from Hughes et al.) [7]	Ulceration (U)	Fistula or abscesses (F)	Stricture (S)		
	0 – not present 1 – superficial fissures (involve squamous lining of anal canal) (a) Posterior and/or anterior (b) Lateral (c) With gross skin tags 2 – Cavitating ulcers (occurs in the upper anal canal or adjacent rectal mucosa) (a) Anal canal (b) Lower rectum (c) With extension to perineal skin (aggressive ulceration)	Subdivided into high or low on the basis of relation to the anorectal ring 0 – Not present 1 – Low or superficial (a) Perianal (b) Anovulvar/anoscrotal (c) Intersphincteric (d) Anovaginal 2 – High (a) Blind supralevator (b) High supralevator (c) High complex (d) Rectovaginal (e) Ileoperineal	0 – Not present 1 – Reversible stricture (a) Anal canal – spasm (b) Low rectum – membranous (c) Spasm with severe pain (no sepsis) 2 – Irreversible stricture (a) Anal stenosis (b) Extrarectal stricture		
AGA classification [6]	Simple	Complex			
	Low (superficial or low intersphincteric or low transsphincteric origin of the fistula tract) Single external opening No pain or fluctuation to suggest perianal abscess No anorectal stricture No rectovaginal fistula No active rectal Crohn’s disease	High (high intersphincteric or high transsphincteric or extrasphincteric or suprasphincteric origin of the fistula tract Multiple external openings Associated with the presence of pain or fluctuation to suggest perianal abscess Rectovaginal fistula Anorectal stricture Active rectal Crohn’s disease			
Fistula Drainage Assessment (FDA) [12]	A perianal fistula is considered to be closed when there is no drainage on gentle compression with a finger				
	Discharge	Pain/restriction of activities	Restriction of sexual activity	Type of perianal disease	Degree of induration
Perianal Crohn’s Disease Activity Index (PDAI). Adapted from Irvine et al. [13]	Range 0–4, where 0 is no discharge and 4 is gross faecal soiling	Range 0–4, where 0 is no activity restriction and 4 is severe pain with severe limitation	Range 0–4, where 0 is no restriction of sexual activity and 4 is unable to engage in sexual activity	0 – No perianal disease or skin tags 1 – Anal fissure 2 – < 3 perianal fistulae 3 – > 3 perianal fistulae 4 – Anal sphincter ulceration	Range 0–4, where 0 is no induration and 4 is gross fluctuance or abscess

Whilst these classification systems focus on anatomical features, others such as the Fistula Drainage Assessment (FDA) and Perianal Disease Activity Index (PDAI) focus on measures to quantify disease activity. The FDA was developed as an outcome measure for the ACCENT study investigating the use of infliximab to treat pCD and relies on a simple examination of a fistula to determine whether it is active, or closed [12]. The PDAI is also widely used in research, measuring disease activity according to five features: discharge, pain/restriction of activities, restriction of sexual activity, type of perianal disease and degree of induration [13]. Although this index was validated in a cohort of 37 patients during its development, it lacks adequate psychometric measurement properties as identified in a systematic review and COnsensus based Standards for the selection of health Measurement INstruments (COSMIN) appraisal by Joshi et al. (manuscript in progress). Additionally, some features such as the degree of induration remain subjective and may require cross-cultural validation.

In complex perianal fistulising Crohn’s disease, cross-sectional imaging, in particular magnetic resonance imaging, is a key component of a thorough assessment. A number of radiological classifications have been developed, including the St James’s University Hospital Classification (see Table 2) [14]. This characterises fistulae on the basis of their location, complexity and involvement of the anal sphincter, elaborating on the Park’s classification by incorporating features such as abscesses and additional tracts that are visible on MR imaging [14]. More recent advances include MRI-based disease activity indices, such as the Van Assche Index (VAI) and the Magnetic Resonance Novel Index for Fistula Imaging in Crohn’s Disease (MAGNIFI-CD), which aim to provide objective assessments of disease activity on the basis of MRI-derived anatomical features. However, their clinical utility remains limited owing to the complexity of scoring, the requirement for gadolinium contrast and their reliance on largely static anatomical parameters, which may reduce sensitivity to subtle changes in fistula activity.

An ideal classification would be biological in nature, identifying with certainty different versions or stages of the disease process according to biological markers. No such classification nor the biological data to produce one currently exist, so pragmatic, phenotype-based classifications remain necessary but require improvement over historical versions.

**Table 2:** St James’s University Hospital radiological classification

Grade	Description
Grade 1	Simple linear intersphincteric fistula
Grade 2	Intersphincteric fistula with abscess or secondary tract
Grade 3	Transsphincteric fistula
Grade 4	Transsphincteric fistula with abscess or secondary tract
Grade 5	Supralevator and/or rectal fistula

### TOpClass classification

**Fig. 2:**
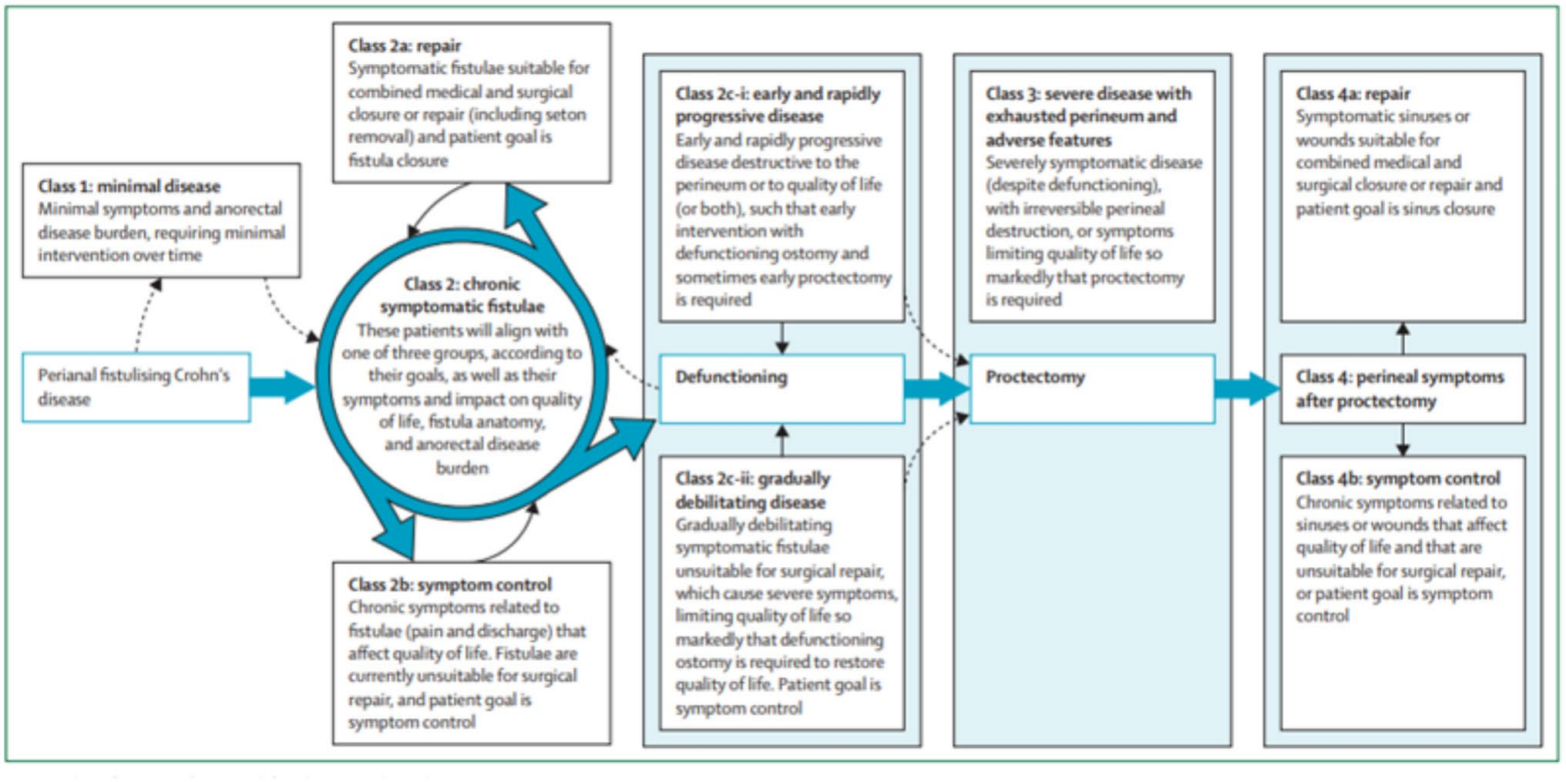
The TOpCLASS classification system for perianal fistulising Crohn's disease. Reproduced with permission from Geldof et al. [8]

The TOpCLASS consortium recently introduced a novel classification system for fistulising perianal Crohn’s disease (pfCD), designed to align patient and clinician goals more effectively by allowing for dynamic movement between classes as disease progression or remission occurs [8]. This classification system, developed by expert consensus and informed by systematic review, heralded an innovative approach categorising patients with fistulising pCD at therapeutically distinct stages through the natural history of the disease [8]. Other key features include the centrality of patient goals, and ability to move between classes, reflecting the chronic nature of the disease and the changing aims of patients as they live with the condition [8]. This represents a pragmatic approach, with clinical descriptors providing greater use in a modern multidisciplinary team (MDT) setting than anatomical descriptors alone. Additionally, the classes allow easier stratification into groups for clinical trials assessing differences in pathogenesis and response to treatment, and recognising the different patient populations and interventions within pfCD but also the different outcomes relevant at different stages of the disease (Fig. 2).

The TOpClass consortium recently published treatment guidance relating to each class, based on background evidence for medical and surgical therapies alongside the practices of MDT members at eight IBD centres in Europe, the USA and Australia [15]. The expert panel voted on a series of new statements, and each centre reviewed a series of case vignettes relating to the classification groups. Position statements for surgical treatment in each class are summarised below (Table 3) [15]:

**Table 3:** Consensus statements on treatment optimisation in perianal Crohn’s disease

TOpClass group	New consensus statements for surgical treatment
Class 1	There is no role for seton insertion or MRI surveillance in the absence of significant fistula symptoms
Class 2a	Criteria for the suitability of surgical repair include the absence of proctitis, anal stricture and florid perianal disease. Anal stricture can be considered a relative contraindication in a well-informed and appropriately consented patient Advancement flap is the most suitable surgical option for patients with a single internal opening and pliable tissues. Ligation of intersphincteric fistula tract (LIFT) is best for patients with thin, transsphincteric tracts without intersphincteric complexity. Fistula plugs or glues are not recommended Fistula tracts with single or multiple internal openings and no undrained perianal collections can be considered for debridement and closure of the internal opening, with or without the use of stem cells Fistula tracts too complex for anatomical repair may be treated by stem cell repair if they meet appropriate criteria Surgical repair attempts should only be considered after optimisation of medical therapy, and usually after seton has been inserted for drainage The removal of setons alone in a medically optimised patient can be considered a repair attempt. Although there is not clear evidence to guide the timing of seton removal, this can be considered after induction therapy
Class 2b	Patients in this category with fistulae with single or multiple openings are candidates for symptom control palliative video assisted anal fistula treatment (pVAAFT) during examination under anaesthesia (EUA)
Class 2ci	Class 2ci (rapidly progressive disease) must be identified quickly, and managed by optimising medical therapy and repeated EUAs until adequate drainage can be achieved. If improvement is not seen within 3–6 months, consider defunctioning stoma. Consider early referral to a high-volume centre
Class 4	There is a need for further research to improve management of these patients and differentiate between the variety of potential therapeutic options. For patients with a persistent sinus, imaging is useful in preoperative planning, monitoring response and identifying underlying pathology, with timing dependent on indication and clinical status. For class 4a, optimisation of the perineal tissues was recommended prior to surgical repair, which might include drainage of the tissues or consideration of excision of the mesorectum, depending on anatomy

The TOpClass classification has not yet been validated in a large prospective cohort. However, the classification system has been retrospectively applied to 96 patients with fistulising pCD [16]. The majority of patients in this cohort were initially classified as class 2b, but around 52.1% of patients changed to a different class. Retrospective classification, particularly in terms of patient goals, presents challenges, and further validation in large prospective cohorts is necessary to improve understanding of how patients may transition between classes. The prospective arm of cohort studies such as Goals, Needs and Determinants Of Multimodal therapy in perianal cRohn’s Fistula (GONDOMAR) may provide insights [17].

### Non-fistulising perianal Crohn’s disease

Although fistulae are the most common manifestation of pCD, population studies indicate that non-fistulizing manifestations are also frequently observed [18]. ‘Fissures’ and ulcers may occur in up to 1/3 patients, abscesses in around half, strictures in 7% and skin tags up to 10% [18, 19] of patients. Haemorrhoids, anal cancer and regional cutaneous manifestations are associated with pCD but probably do not represent actual pCD manifestations, and data around them are limited.

Non-fistulising disease is also included in a number of the classification systems that are in widespread use for fistulising pCD, such as the Cardiff–Hughes classification (33) or the Buchmann classification [20], which classify largely on the basis of anatomical or morphological features. Other indices that we use frequently to assess disease activity, such as the Perianal Disease Activity Index [13], were validated on patients with both fistulising and non-fistulising pCD, and include elements such as fissures and skin tags.

Several classification systems focussing on specific features of non-fistulising pCD have been described. In 1975, Greenstein categorised anorectal strictures in Crohn’s by length, subdividing into annular < 2 cm strictures, tubular > 2 cm strictures and strictures due to post-ileostomy atrophy [21]. More recently, for a small paediatric case series, strictures were defined as severe or non-severe, with the former being unable to tolerate a colonoscope or digital rectal examination [22]. In terms of ulcerating disease, Horaist et al. performed an a expert consensus process to define and classify common pCD lesions [23], classifying ulcers by depth, extension and location. Fissuring and ulcerating disease has been classified as simple or complicated, with complicated disease defined by the involvement of both sphincters and requiring operative management [24]. The AGA technical review classified skin tags broadly into two types, with type 1 being large, hard and cyanotic, and type two being soft, flat and painless [6].

The wide variety of features that are included in classification systems for non-fistulising pCD emphasises the lack of consensus around which features are related to pCD and which are common proctologic conditions that may present in the CD population. Key considerations for any future classification system would include characteristic features, the interaction between true pCD manifestations and common proctological problems, how to define severity, boundaries and movement between classes, and how to capture the relationship between fistulising and non-fistulising disease. This may enable future trials to capture these manifestations of pCD more effectively and provide further insight about treatment outcomes and prognosis.

## Retrospective classification of previous clinical trials

Clinical trials exploring the medical and surgical management of pCD often involve diverse patient cohorts, but they typically provide limited detail on individual patient goals. The PISA II trial was a landmark study that sought to address this balance by incorporating a patient-preference treatment arm into its methodology [25]. The TOpClass classification enables more effective organisation of patients with pCD into homogeneous groups for inclusion into future studies [8]. To date, the TOpClass classification has not been used in the context of a clinical trial evaluating treatments for pCD.

A scoping review of clinical trials in pCD was performed to understand how the TOpClass classification relates to existing evidence. The National Institutes of Health database was searched via ClinicalTrials.gov using the search term “Perianal Crohn’s disease”. The search was limited to trials first posted from 1 January 2000 to 18 June 2024. Only phase 2, 3 and 4 trials were included. The search was conducted on 18 June 2024. Trials that were not yet recruiting, were terminated without results or involved children (0–17 years) were excluded. Twenty-four key studies investigating the management of pCD were identified, with a further three studies identified via citation search. Eight studies were excluded in total because they had been terminated without publishing, were not yet recruiting, did not relate to Crohn’s or did not relate to perianal fistulae. Details of the patient cohorts reported in the included trials were re-assessed by two reviewers, including one senior author, to determine how and whether they could be classified using the TOpClass classification.

Of the 19 studies, 4 studies described cohorts consistent with class 2a. Seven were classified as class 2a or 2b, but were unable to subdivide further. Seven were consistent with class 2a, b, or c but were unable to be classified any further. One study was classified as class 2a or 2c. Overall, eight studies did not specify whether patients with diverting stomas were excluded from the patient cohorts.

Four studies described patient cohorts consistent with the TOpClass 2a group. These were, PISA II (Anti-Tumour Necrosis Factor vs Surgical Closure following Anti-TNF), the ADMIRE trials (adipose-derived mesenchymal stem cells) and a trial investigating darvadstrocel (expanded adipose stem cells – eASC) [25, 26]. Of these, ACCENT II and the BM–MSC trial did not specify whether patients with diverting ostomies were excluded so there may have been patients in class 2c in these cohorts. Stomp II did not exclude patients with stoma, and therefore patients in this cohort with stoma would be grouped into class 2c (NCT04847739). In all these cohorts, it was assumed that, by giving informed consent to take part in these clinical trials, they were indicating a preference for (demonstrating that their goals aligned with) the interventions being investigated. PISA I is a good example of a trial in which the interventions did not all align to a single patient goal [27], which was probably a factor in difficulty in recruitment.

Seven studies described patient cohorts that could be classified as either class 2a or 2b, but it was not possible to subdivide further. These included CHARM, DIVERGENCE 2, Fuzion CD, ExoFlo, USTAP, ENTERPRISE and a study investigating the use of therapeutic dose monitoring in infliximab [28, 29]. These studies included a patient cohort of active fistulae, where patients with stomas were excluded, but the range of fistulae complexity reported would include both fistulae suitable and not suitable for repair.

A total of seven studies described patient cohorts with active fistulae but did not provide adequate anatomical information or information regarding patient goals to classify any further. These trials included ACCENT, ADAFI, CHOICE and studies investigating the use of Fibrin Glue, Topical metronidazole, Bone Marrow Derived Stem Cells (BM-MSC), and the use of endoscopic ultrasound to administer Humira [30–36]. None of these studies specified whether patients with stomas were included or excluded from their cohorts.

Within the limitations of a retrospective analysis of registered studies, these findings demonstrate the heterogeneity of patient cohorts included in previous clinical trials investigating treatments for fistulising pCD. In particular, this indicates that, whilst the I and C of Patient/Population, Intervention, Comparison, Outcome (PICO) have been chosen carefully and represent the focus of a trial’s design, the P and O have often not been considered in adequate detail. Patients with substantially different disease phenotypes in terms of complexity and suitability for different treatments are frequently grouped together.

For example, the precise impact of defunctioning ostomies on the microbiome and subsequent inflammation in the distant bowel remains unclear. However, it is reasonable to suggest that patients with stomas may respond differently to novel treatments compared with those without, just as might be the case in those with fistulae too complex to repair, or active anorectal disease which precludes repair. Reporting results separately for these groups, which the TOpClass classification would facilitate, could provide more meaningful insights.

Class 2a patients (fistulae suitable for repair) who are willing to undergo surgery are ideal for a trial of surgical repair versus seton removal alone, for example, or surgical repair with or without therapeutic drug monitoring – supported combination medical therapy. Class 2b patients might be included in trials in which the outcomes of interest are quality of life improvement and downstaging to class 2a. Those class 2b patients with high complexity fistulae, or uncontrolled proctitis, might enter trials which compare open versus VAAFT-assisted fistula rationalisation (downgrading a complex fistula not amenable to repair), or different advanced medical therapies, to bring about anatomical or biological rationalisation, respectively.

**Fig. 3:**
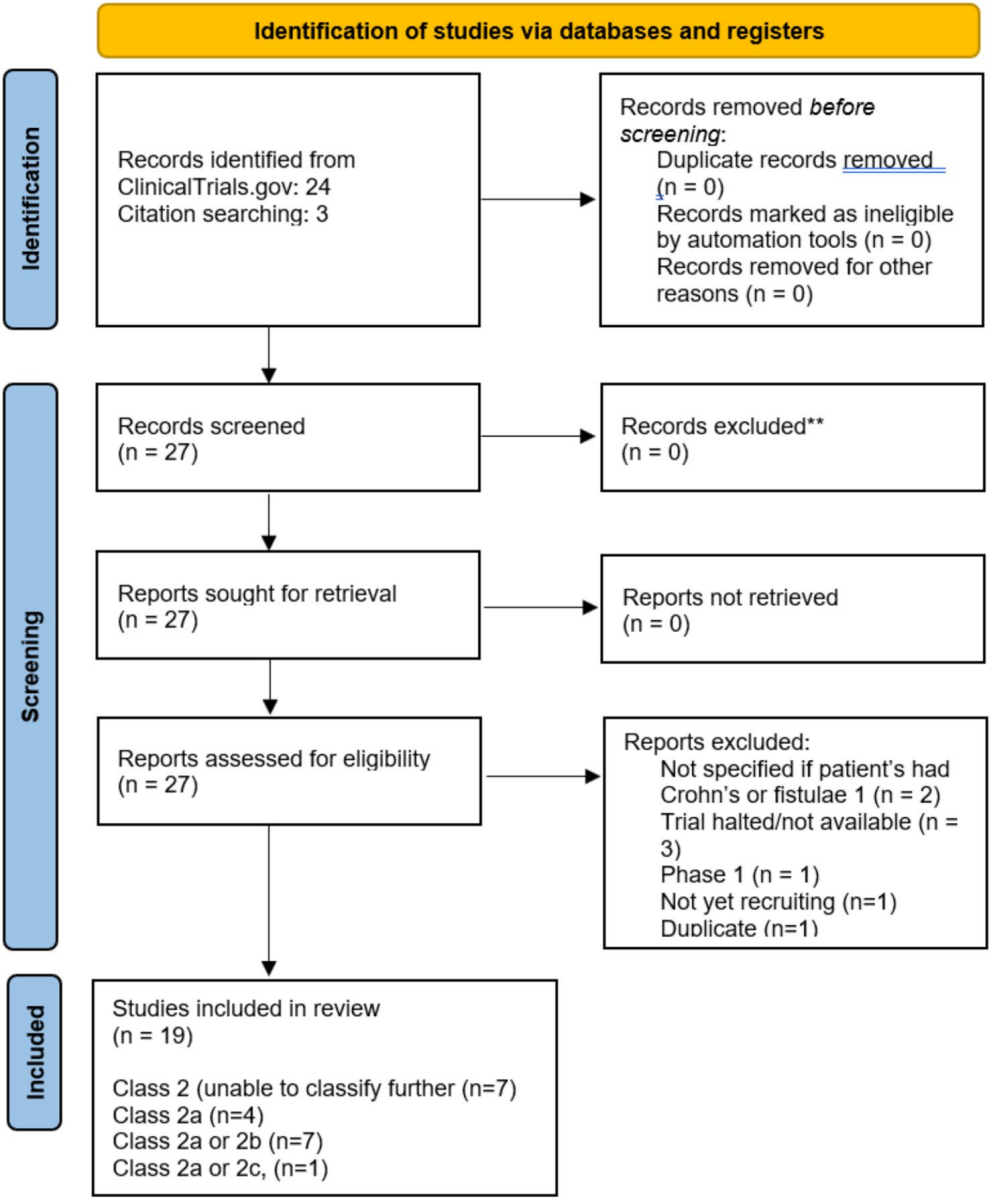
PRISMA diagram for scoping review

Researchers can then consider the various options for trial inclusion criteria, interventions and outcomes of interest according to class, facilitating a much wider and richer landscape for studying pfCD at every stage of the disease (Fig. 3; Table 4).

**Table 4:** Summary of scoping review

Trial name	Author	Methodology	Therapeutic agent	Patient cohort	Patients with defunctioning ileostomy/colostomy included	TOpClass classification
ACCENT II	Sands et al. [31]	Randomised double-blind placebo-controlled study	Infliximab	> 18 years of age, with single or multiple draining fistulae, including perianal, enterocutaneous, and rectovaginal fistula. Numbers of active fistulae ranged from 1 to > 5. Setons were removed	Not specified	Class 2 – unable to subdivide further
PISA II	Meima et al. [37] NCT06441526	Patient-preference randomised controlled trial	Infliximab/adalimumab or anatomic surgical closure – LIFT or advancement flap	> 18 years of age, active high perianal fistula and single internal opening	Excluded	Class 2a (anatomical) repair
CHARM	Colombel et al. [28]	Randomised double blind placebo controlled trial	Adalimumab	18–75 years of age, with enterocutaneous or perianal fistulae	Excluded	Class 2a or 2b – unable to subdivide further. May also have included class 1
ADMIRE CD	Panes et al. [26] NCT01541579	Phase 3 randomised double-blind placebo controlled trial	Adipose-derived mesenchymal stem cells (Cx601)	> 18 years with complex perianal fistulae (high intersphinteric/transsphincteric or suprasphincteric, at least two external openings, actively draining)	Excluded	Class 2a (anatomical or non-anatomical repair)
ADAFI	Dewint et al. [33] **NCT00736983**	Randomised double-blind placebo controlled trial	Adalimumab combined with ciprofloxacin in comparison with adalimumab monotherapy	Patients 18–70 years of age with active fistulising pCD. Number of fistulas draining ranged from 1 to > 3, with the majority having 1 or 2	Not specified	Class 2 – unable to subdivide further
CHOICE	Lichtiger et al. [32]	Open label trial	Adalimumab in patients who failed prior infliximab therapy	Patients 18–75 with moderate to severe CD who have failed prior infliximab therapy. Patients had at least one draining fistula, with the majority being perianal	Not specified	Class 2 – unable to subdivide further
DIVERGENCE II	Reinisch et al. [29] NCT03077412	Phase 2 double blind multicentre trial	Filgotinib	18–75 years, moderately to severely active fistulising perianal Crohn’s disease. Fistulas had 1–3 openings and setons were removed. 80–100% complex fistulae – multiple tracts, trans-, extra- or suprasphincteric tracts	Not specified, but absent from results	Class 2a or 2b – unable to subdivide further
Fuzion CD	Unpublished, recruiting (NCT05347095)	Multicentre phase 3 randomised placebo-controlled study	Guzelkumab	Patients > 18 years of age with at least one active draining fistula	Excluded	Class 2a or 2b – unable to subdivide further
ExoFlo	Unpublished NCT05836883	1b/2a single blind randomised controlled trial	ExoFlo (mesenchymal stem cell derived product	Patients 18–75 with single or multiple tracts who have failed previous surgical intervention, are not candidates or are not willing to have surgical intervention	Not specified	Class 2a or 2b – assuming no patients with stomas recruited
STOMP 2	Dozois et al. [38] NCT04847739	Phase 2	Mesenchymal stem cells incubated in a matrix plug	Included patients had a single fistula tract with one internal opening and one external opening. Patients with a stoma were not excluded. Participants with complex fistulas such as horseshoe configuration, blind sinuses, or more than one internal opening were excluded	Not excluded	Class 2a or 2c
USTAP	Unpublished. Currently recruiting NCT04496063	Randomised double blind placebo controlled trial	Ustekinumab	Patients with at least one active perianal fistula tract	Excluded	Class 2a or 2b – unable to subdivide further
Fibrin glue	Grimaud et al. [35] **NCT00723047**	Multicentre, open label randomised controlled trial	Fibrin glue injections	Patients with at least one perianal fistula. With approximately equal proportions of simple and complex fistulae	Not specified	Class 2 – unable to subdivide further
Topical metronidazole	Maeda et al. [36] NCT00509639	Randomised controlled trial	Metronidazole ointment	74 patients with active symptoms. No further information given regarding the number and nature of the fistulae	Excluded if stoma for less than 6 months	Class 2 – unable to subdivide further
Admire CD 2	Unpublished NCT03279081	Phase 3 randomised controlled trial	Adipose-derived mesenchymal stem cells (Cx601)	Patients with complex active fistulae with two internal openings and a maximum of three external openings. High intersphincteric, transphincteric or suprasphincteric, with associated abscesses	Excluded	Class 2a, suitable for anatomical or non-anatomic repair
Bone marrow derived mesenchymal stem cells	Molendijk et al. [34] NCT01144962	Randomised controlled trial	Bone marrow derived mesenchymal stem cells	1–2 internal openings and 1–3 fistula tracts. Complex fistulae (> 2 external openings) were excluded	Not specified	Class 2, unable to subdivide further
Enterprise	Scwartz et al. [39] NCT02630966	Randomised double blind trial	Vedolizumab (two different regimes)	Adult patients with 1–3 draining fistulae. Patients with diverting ostomies and more than three fistulae were excluded/ Majority had one draining fistulae	Excluded	Class 2a or b – The majority of patients are likely to be class 2a suitable for anatomical repair, but without further anatomical detail this cannot be confirmed
Endoscopic US guided treatment with Humira	Wiese et al. [30] NCT00517296	Randomised trial	Rectal EUS to guide treatment using adalimumab	Complex disease, but not defined in terms of AGA technical review definitions	Not specified	Class 2 – unable to classify further
Darvadstrocel	Furukawa et al NCT04118088	Phase 3, open label single arm trial	Darvadstrocel	Active, high intersphincteric, transphincteric or suprasphincteric fistulae. Fistulae had at least two external openings or associated fluid collections	Excluded	Class 2a
TDM infliximab	Unpublished, currently recruiting (NCT06051253)		Therapeutic drug monitoring of infliximab	At least one actively draining fistula	Excluded	Class 2a or b – unable to subdivide further

## Conclusions

This narrative review provides an overview of the classification systems that have been described in the literature to classify fistulising pCD and the described recent advances such as the TOpClass classification system. The re-categorisation of existing clinical trials using the TOpClass classification highlights the practical benefits such a system can deliver in both clinical and research settings. Finally, this article has outlined several existing classification systems in use for non-fistulising pCD and highlighted the need for a novel classification. The many advances in the treatment of pCD in terms of advanced medical therapies, novel procedures such as VAAFT, fistula laser closure (FiLaC) and regenerative medicine are encouraging for the future. There is a need to ensure that there is consistency in how we define, classify and report outcomes in clinical trials.

To return to Hippocrates, it is true that “that which is used, develops… that which is not used, wastes away”. Ultimately, the test of any new clinical measure or classification system is whether healthcare professionals in busy wards and clinics around the world find it to be a useful adjunct to their daily practice, and whether researchers adopt it as they design the next iteration of clinical trials in pfCD. The benefits of the TOpClass classification system will lie in its integration into shared decision-making processes within joint surgical–medical clinics and IBD MDTs, and its role in re-classifying and stratifying patients in clinical trials. It is expected that the TOpClass classification will continue to develop and evolve through active use and improvement in the future, until a true biological classification can replace it.

## Data Availability

Data sharing not applicable to this article as no patient datasets were generated or analysed during the current study. No datasets were generated or analysed during the current study.

## Declarations

### Provenance and peer review

Not commissioned, externally peer reviewed.
